# Monozygotic dichorionic-diamniotic pregnancies following single frozen-thawed blastocyst transfer: a retrospective case series

**DOI:** 10.1186/s12884-020-03450-5

**Published:** 2020-12-10

**Authors:** He Li, Tingting Shen, Xiaoxi Sun

**Affiliations:** 1 Shanghai Ji Ai Genetics and IVF Institute, Obstetrics and Gynecology Hospital, Fudan University, Shanghai, 200011 China; 2 Key Laboratory of Female Reproductive Endocrine Related Diseases, Obstetrics and Gynecology Hospital, Fudan University, Shanghai, China

**Keywords:** Assisted reproductive technology, Monozygotic dichorionic-diamniotic twinning, Single blastocyst transfer

## Abstract

**Background:**

The primary aim of the study is to report cases of monozygotic dichorionic-diamniotic (DC-DA) pregnancies after single frozen-thawed blastocyst transfer.

**Methods:**

This is a retrospective case series. All single frozen-thawed blastocyst transfer cycles performed between June 2013 and December 2018 at the Shanghai Ji Ai Genetics and IVF Institute, Obstetrics and Gynecology Hospital, Fudan University, Shanghai, China, were reviewed retrospectively. We included frozen embryo transfer (FET) cycles which clinical pregnancy was confirmed with multiple gestational sacs showed on ultrasonography at around 6 to 7 weeks of gestation. We then conducted an in-depth analysis to further exclude cases which contained newborns of different genders or natural FET cycles.

**Results:**

Five thousand four hundred fifteen frozen-thawed single blastocyst transfer cycles were preformed between June 2013 and December 2018 at the Shanghai Ji Ai Genetics and IVF Institute, Obstetrics and Gynecology Hospital, Fudan University, Shanghai, China. Of these, fourteen women underwent a single blastocyst transfer and then achieved clinical pregnancy with an ultrasound diagnosis of multi-chorionic pregnancy. With one natural cycle FET excluded, we finally included thirteen single blastocyst transfer cycles performed in down-regulated controlled FET or hormone replacement FET, in which the possibility of concurrently spontaneous pregnancy was extremely small. These included 13 cases reveal the phenomenon of monozygotic DC-DA twinning after single blastocyst transfer, which challenges the classical theory that only monochorionic pregnancy could happen after 3 days of fertilization.

**Conclusion:**

This case series suggest that single blastocyst transfer could result DC-DA pregnancies during IVF treatment.

## Background

When a single embryo divides into two after fertilization defines the type of twin pregnancy. There is a classical theory presented by Corner about the timing of embryo division and twin pregnancy development: within 3 days of fertilization, dichorionic-diamniotic (DC-DA) twins; between 4 and 8 days, monochorionic-diamniotic (MC-DA) twins; and between 9 and 12 days, monochorionic-monoamniotic (MC-MA) twins; and rarely, after 12 days, conjoined twins [1, 2].

Assisted reproductive technology (ART) has been associated with multiple gestations as a result of transfer with more than one embryos, which may develop into DC-DA twins. However, the frequency of monozygotic twinning after ART also increases, which varies from 0 to 13.2% compared with 0.4% of live births in spontaneous conception population [3, 4]. Although the specific mechanism of the increased risk of monozygotic twinning with in vitro fertilization (IVF) is controversial, researchers have proposed a lot of risk factors including extended embryo culture, female age and certain IVF procedures, especially those related to micromanipulation of the zona pellucida as intracytoplasmic sperm injection (ICSI) or assisted hatching (AH), embryo biopsy and embryo cryopreservation [3–8].

The information on monozygotic DC-DA twinning after single blastocyst transfer is extremely limited. As it differs with the deep-rooted multiple pregnancy theory that single blastocyst transfer should only lead to MC-DA or MC-MA twinning, more studies and further investigation are needed. Many DC-DA twinning are suspected to be the results of either transfer of more than one embryo or less common, spontaneous pregnancies at the same time [9]. DC-DA monozygotic twinning rate is underestimated because genetic testing of offspring is rarely performed [10]. Therefore, we collected and analyzed data of thirteen cases of monozygotic DC-DA twinning after single blastocyst transfer in down-regulated or hormone replacement frozen embryo transfer (FET) cycles.

## Material and methods

We retrospectively reviewed all frozen thawed single blastocyst transfer cycles performed between Jan 2013 and December 2018 at the Shanghai JiAi Genetics & IVF Institute. This study was approved by the Ethics Committee of Assisted Reproductive Medicine in Shanghai JiAi Genetics & IVF Institute (JIAI E2020–02).

FET cycles were analyzed when clinical pregnancy was confirmed alone with one or more gestational sacs showed on ultrasonography at around 6 to 7 weeks of gestation. We further identified all patients with two or more gestational sacs noted on initial ultrasound, suggesting possible multi-chorionic monozygotic pregnancies. We then conducted an in-depth analysis to further exclude cases which contained newborns of different sexes or natural FET cycles, in order to rule out the possibility of dizygosity. Demographic data and IVF treatment information including obstetrical and neonatal outcome data of the included cases were summarized and analyzed. Moreover, we compared the demographic and IVF-FET cycle characteristics between monochorionic and dichorionic twining group. Comparison of quantitative variables were performed using Student’s t-test, while categorical variables were compared using a χ2 analysis. All statistical analyses of the data were performed using the SPSS program V.21.0 (SPSS), and a *P* value < 0.05 was considered statistically significant.

Women underwent IVF, ICSI or preimplantation genetic testing (PGT) treatment in the center according to clinical indications. Protocols of controlled ovarian stimulation included: gonadotropin-releasing hormone (GnRH) antagonist protocol, short or long GnRH agonist protocol, and clomiphene citrate (CC) + human menopausal gonadotropin (hMG) or follicle-stimulating hormone (FSH) protocol.

Oocyte retrieval was performed 34-36 h after human chorionic gonadotropin (hCG) or GnRH agonist trigger under transvaginal ultrasound guidance. Obtained oocytes were fertilized using either conventional IVF or ICSI as clinically indicated, and incubated in fertilization media (Vitrolife, Sweden). Fertilization was judged by the appearance of two pronuclei and a second polar body at 16–18 h after IVF or ICSI. Fertilized zygotes were grown to the blastocyst stage in sequential culture media (G1 and G2, Vitrolife).

Assisted hatching was performed on D3 embryo with an 18 μm hole made in the zona pellucida of the embryos. PGT biopsy was performed on day 5 or day 6 embryo. Approximately 3–5 trophectoderm cells were biopsied using a pipette and placed into polymerase chain reaction (PCR) tubes. Then the cells were either directly used for Whole genome amplification (WGA) or cryopreservation for later WGA. All the testing experiments and data analysis were completed in Ji Ai local genetic laboratory.

Endometrial preparation for FET was achieved by either hormone replacement treatment (HRT) or down-regulated HRT-FET. For HRT-FET, on day 3 of the menstrual cycle, estradiol valerate (E2, Progynova, Schering AG, Berlin, Germany) was commenced 4 mg daily for 10–12 days. When the thickness of the endometrium reaches at least 7 mm on pelvic ultrasound scanning, progesterone in oil (80 mg) was added. Ultrasound was performed not only to evaluate endometrial lining, but also to confirm no dominant follicles in bilateral ovaries. For down-regulated FET, GnRH agonist was usually given in the mid luteal phase (day 21) of the menstrual cycle. Pituitary down-regulation was confirmed on the second or third day of the expected next menstruation. If baseline levels have been reached and the ovaries are quiescent on pelvic scanning, HRT with estradiol valerate was started as described above.

Blastocyst transfer was scheduled on the sixth day of starting intramuscular progesterone. Single blastocyst with the best morphology was transferred under transabdominal ultrasound guidance using a soft catheter. After thawing, embryo score was assessed according to Gardner morphological criteria [11], on the basis of the degree of expansion and the development of the inner cell mass and trophectoderm. Serum hCG level was checked 14 days after FET. All hormone therapy was stopped if the serum hCG level was negative. Pregnant women continued the hormonal therapy until 12 weeks of gestation.

## Results

Five thousand four hundred fifteen single blastocyst FET cycles were performed between June 2013 and December 2018 at our infertility center and 2510 (46.4%) resulted in a clinical pregnancy. From the 1510 clinical pregnancies, 43 (2.8%) were monozygotic twinning (MZT) pregnancies. 14 (0.9%) of total pregnancies were monozygotic DC-DA pregnancies which had two gestational sacs on 6–7 gestational weeks` ultrasound examination. 29 (1.9%) of total clinical pregnancies were monozygotic monochorionic pregnancies. Excluding one natural cycle FET, finally we included 13 women who were in down-regulated controlled FET or hormone replacement therapy FET (with FET cycle ultrasound documenting absence of spontaneous ovulation), in which concomitant spontaneous pregnancy could not happen. The incidence rate of monozygotic DC-DA pregnancies was 0.5% (13/2510) after single blastocyst FET.

**Fig. 1:**
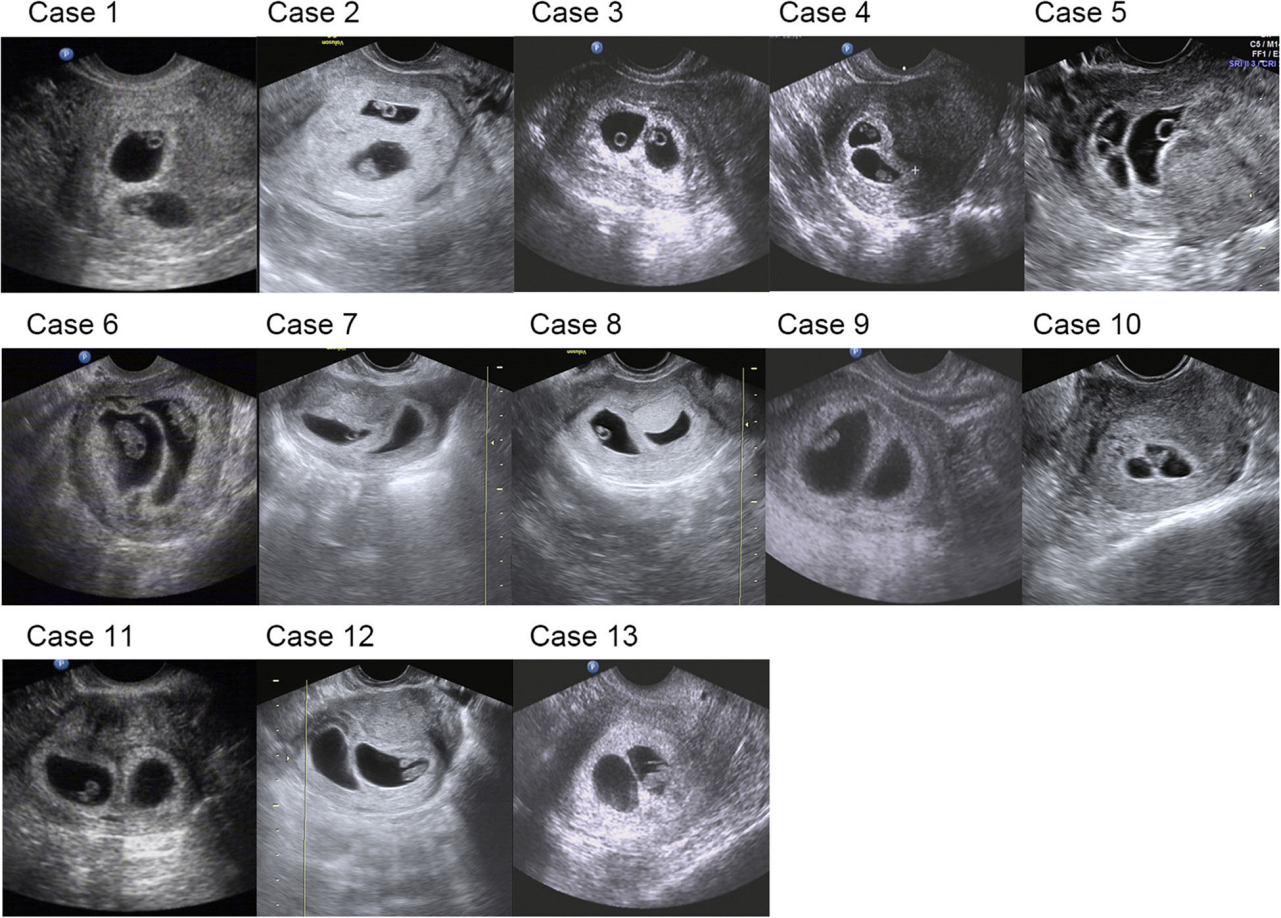
The images of the initial ultrasound at gestational 6–7 weeks. Case 1 to 6: gestational 6–7 weeks` ultrasound with 2 gestational sacs and 2 fetal poles. Case 7 to 13: gestational 6–7 weeks` ultrasound with 2 gestational sacs and 1 fetal pole

Demographic data about these 13 patients are showed in Table 1. ART treatment information are showed in Table 2. Pregnancy outcomes are showed in Table 3. Demographic and IVF-FET cycle characteristics between monochorionic and dichorionic twining group were comparable, which was showed in Table 4. All the included thirteen patients had at least one reported risk factors associated with monozygotic twinning, including the procedure of ICSI (9/13), assisted hatching (5/13), and blastocyst transfer (13/13). PGT was performed in 5/13 patients. Figure 1 shows the initial ultrasound confirming dichorionic-diamniotic twinning at 6 to 7 weeks of gestation.

**Table 1:** Demographic characteristics

	Age (years)	BMI (kg/m2)	Gravidity	Parity	Diagnosis	Antral follicle count	No. of prior IVF attempts	No. of prior D3 embryos transferred	No. of prior blastocysts transferred
Case 1	31	21.23	4	0	Recurrent miscarriage, male reciprocal translocation	27	0	0	0
Case 2	38	24.97	1	0	Tubal factor and male factor	13	2	2	0
Case 3	33	17.97	2	0	Tubal factor	24	0	0	0
Case 4	34	17.19	0	0	Male factor	18	0	0	1
Case 5	41	21.26	3	1	Tubal factor and male factor	13	0	0	0
Case 6	33	19.92	1	1	PCOS and male factor	19	0	4	0
Case 7	28	20.51	0	0	Endometriosis	8	0	0	0
Case 8	39	23.31	3	0	Recurrent miscarriage	18	0	0	1
Case 9	38	19.92	3	0	SNM1 mutation	24	0	0	3
Case 10	30	18.59	0	0	Male robertsonian translocation	16	0	0	0
Case 11	42	22.83	2	1	Unexplained infertility	12	0	0	0
Case 12	43	21.97	2	0	Recurrent miscarriage	8	0	0	1
Case 13	32	25.39	2	1	Tubal factor	29	0	0	0

*PCOS* polycystic ovarian syndrome, *SNM1* survival motor neuron gene

**Table 2:** IVF cycle characteristics

	COH protocol	Gonadotropin injection (units)	Days of stimulation	# of oocytes retrieved	# of blastocysts	Fertilization method	AH	PGT	Endometrial preparation	Embryo day at transfer	Grade of blastocyst at thaw	Endometrial thickness in FET (mm)
Case 1	Antagonist	975	9	32	3	ICSI	Yes	Yes	HRT	D5	B5BC	9
Case 2	Short agonist	3225	12	17	1	ICSI	No	No	HRT	D5	B3BB	8
Case 3	Antagonist	750	9	11	4	IVF	No	No	HRT	D5	B4BA	10
Case 4	CC + HMG	850	10	21	9	ICSI	No	No	Down-regulated	D5	B5BA	9
Case 5	Short agonist	1800	8	14	7	ICSI	No	No	HRT	D5	B4AB	8
Case 6	Antagonist	1000	8	18	5	ICSI	No	No	Down-regulated	D5	B3CB	9
Case 7	Long agonist	3300	11	7	1	IVF	No	No	Down-regulated	D5	B4BB	11
Case 8	CC + HMG	1125	9	9	3	ICSI	Yes	Yes	HRT	D5	B5BB	7
Case 9	Antagonist	1450	10	31	16	ICSI	Yes	Yes	Down-regulated	D5	B5CB	9
Case 10	Short agonist	1350	9	21	4	ICSI	Yes	Yes	Down-regulated	D5	B5BC	11
Case 11	CC + HMG	1800	9	6	3	IVF	No	No	Down-regulated	D5	B4BB	11
Case 12	CC + HMG	2250	9	7	3	ICSI	Yes	Yes	Down-regulated	D6	B6AB	8
Case 13	Antagonist	1987.5	10	28	5	IVF	No	No	HRT	D5	B5BC	7

**Table 3:** Pregnancy outcomes

	β-hCG (mIU/mL)	# of gestational sacs	# of fetal poles	Pregnancy outcome	Gestation at delivery (weeks + days)	Birth weight (g)	Gender of newborn(s)	Mode of delivery	Obstetric complications	Neonatal complications
Case 1	979	2	2	2 live birth females	38 + 3	2900/3300	Female/Female	Elective caesarean	Gestational hypertension	
Case 2	1330	2	2	2 live birth males	36	2250/2360	Male/Male	Emergency caesarean		
Case 3	980	2	2	2 live birth females	39 + 1	3000/3010	Female/Female	Elective caesarean		
Case 4	731	2	2	2 live birth males	31 + 2	1500/1650	Male/Male	Emergency caesarean		One newborn had necrotizing enterocolitis and cured by surgery.
Case 5	606	2	2	vanishing twin, 1 liveborn singleton	38 + 5	3150	Female	Elective caesarean	Marginal placenta previa	
Case 6	467	2	2	vanishing twin, 1 liveborn singleton	39 + 3	3150	Female	Elective caesarean		
Case 7	190	2	1	1 liveborn singleton	40 + 4	3850	Male	Vaginal delivery		
Case 8	1367	2	1	1 liveborn singleton	40 + 1	3000	Male	Vaginal delivery	GDM	
Case 9	1370	2	1	1 liveborn singleton	38 + 5	3350	Male	Elective caesarean		
Case 10	582	2	1	1 liveborn singleton	39	3000	Male	Elective caesarean	Gestational hypertension	
Case 11	1370	2	1	1 liveborn singleton	39	3080	Male	Elective caesarean		
Case 12	1289	2	1	1 liveborn singleton	38	3000	Female	Elective caesarean	GDM	
Case 13	2055	2	1	1 liveborn singleton	36 + 3	3150	Male	Vaginal delivery		

*GDM* gestational diabetes mellitus

**Table 4:** Demographic and IVF-FET cycle characteristics in monochorionic and dichorionic twining group

	Monochorionic	Dichorionic	***P***-value
	(***n*** = 29)	(***n*** = 14)	
Age at IVF (years)	34.7 ± 4.5	35.4 ± 4.7	0.674
Body mass index (kg/m^2^)	22.4 ± 3.8	21.2 ± 2.4	0.301
Primary infertility	12 (41.4)	5 (35.7)	0.722
Fertilization method			
Conventional IVF	12 (41.4)	5 (35.7)	0.722
ICSI	17 (58.6)	9 (64.3)	
Assisted hatching			
No	16 (55.2)	8 (57.1)	0.903
Yes	13 (44.8)	6 (42.9)	
PGT			
No	17 (58.6)	8 (57.1)	0.927
Yes	12 (41.4)	6 (42.9)	
Embryo day at transfer			
Day 5	23 (79.3)	12 (85.7)	0.613
Day 6	6 (20.7)	2 (14.3)	
Endometrial preparation			
Natural cycle	2 (6.9)	1 (7.1)	0.976
HRT or down-regulated	27 (93.1)	13 (92.9)	
Endometrial thickness in FET (mm)	9.0 ± 1.1	8.9 ± 1.4	0.856

Data presented as mean ± SD or n (%)

## Discussion

Since the first case of monozygotic twinning associated with IVF was reported in 1984 [12], the incidence of MZT following assisted reproduction has been continually rising [13]. The development model of monozygotic twinning was described by Corner for the first time in 1955 and has become accepted as golden rule, which is now often published in textbooks and literature. A monozygotic DC-DA twining is assumed to develop when a cleavage embryo splits within the first 3 days after fertilization, before the inner cell mass cells differentiate [14]. As Herranz writes, the theory was quickly accepted due to Corner’s prestige, the internal logic of the model, and the convincing nature of his graphic depiction [15]. The universally accepted idea about the various modes of monozygotic twinning (addressed as `the mode`) is based on few experimental data, because embryo experimentation must meet restrictions in humans [16]. However, our study reported 13 single blastocyst FET cases resulted monozygotic DC-DA twinning, which challenged the golden rule.

In our retrospective study examining a large cohort of single blastocyst FETs, we report a 2.8% MZT pregnancy rate following single blastocyst FET, comparable to previous studies [5, 6]. Most of the MZT pregnancies were monochorionic, similar to the previous report [6]. The incidence rate of monozygotic DC-DA pregnancies was 0.5% (13/2510) after single blastocyst FET in our study. Most previous studies of single blastocyst transfer resulted in DC-DA twins are case reports. A recent study reported four cases of single blastocyst transfer resulted in monozygotic DC-DA twins in down-regulated controlled FET cycles, the incidence rate of monozygotic DC-DA pregnancies was 0.3% (4/1181) after single blastocyst FET [17], which was comparable with our study. Other case reports stated atypical hatching would lead to monozygotic twinning after single blastocyst transfer [2, 18, 19]. Another research including 4976 clinical gestations showed that they had never observed an embryo division in half before the blastocyst stage during over 15 years of IVF treatment and laboratory experience [14]. Alone with our findings, the popular credo of chorionicity simply based upon the day of embryonic development must be reevaluated. Moreover, there must be an underestimation of the rate of monozygotic DC-DA twins associated with IVF treatment, because monozygotic multiple pregnancies may not be noticed if more than one embryo are transferred. Since all the published studies are retrospective or case reports with small sample sizes and lack of fetal or neonatal genetic analyses, which makes it difficult to draw concrete conclusions against the long believed dogma and needs further research.

The mechanism of monozygotic division is still unknown. Micromanipulation of the zona pellucida during ICSI, embryo biopsy, and assisted hatching has been reported to be risk factors associated with monozygotic pregnancy during IVF treatment in a lot of studies [9, 19, 20]. According to the most popular theory, AH might increase the incidence of the inner cell mass split and two fetal plates consequently develop [19]. While some other studies show opposite results, that embryo manipulation (ICSI, assisted hatching, embryo biopsy) do not increase the risk of monozygotic pregnancies [9, 21]. In our 13 cases, possible previously reported risk factors related to the incidence of monozygotic multiple pregnancy were embryo biopsy for PGT, AH, ICSI and extended culture. Both AH and embryo biopsy were performed in 5 of the 13 cases; ICSI was done in 9 of the 13 cases in the study.

Single blastocyst transfer is recommended in many countries as it has a favorable prognosis for live-birth as well as low multiple pregnancy. But extended culture may play a role in the development of MZT. Transferring embryos at blastocyst stage exposes the embryo to extra time in the in-vitro environment and may have some effects on the embryo and therefore increase the chance of embryo division. A retrospective analysis showed a 5.6% MZT pregnancy rate in the blastocyst transfer group compared with 2% in the cleavage embryo group [22]. The mechanism is speculated to be long exposure to low levels of calcium might harm the Intracellular stabilization and consequently lead to the division of the inner cell mass [3, 19]. Our study showed a 2.8% MZT pregnancy rate following single blastocyst FET, which was lower than the above study. While another study showed no increase in multiple pregnancies relative to the embryo stage in either the DC-DA or MC-DA twinning [23]. Risk factors for embryo division remain controversial and more researches are needed to answer the question.

A strength of our study is that it includes only down-regulated and HRT single frozen blastocyst transfer, with ultrasound confirming no ovulation during endometrial preparation in FET cycle to rule out the possibility of dizygotic DC-DA twinning. To the best of our knowledge, this is the largest case series about monozygotic multichorionic twining after single blastocyst transfer. One limitation of our study is that monozygosity was not verified by the genetic analysis of the offspring, which is thought to be the gold standard. Therefore, in the future study, we should pay more attention to confirm monozygosity with genetic analysis which is very important and should not be neglected. Another limitation is that we do not routinely take pictures of the transferred embryo on the FET day, so we lack the data of the 13 transferred blastocysts of our included cases.

## Conclusion

In conclusion, we reviewed 13 cases found among 5415 single blastocyst FET cycles resulted in a monozygotic DC-DA gestation. This is the largest case series showed that single blastocyst transfer could result monozygotic DC-DA pregnancies during IVF treatment which challenged the accepted theory. Patients should be informed of a possible increased risk of monozygotic multiple pregnancies after single blastocyst FET. The frequency and mechanism of how the monozygotic multichorionic pregnancy occurs after single blastocyst transfer is still unknown. Further studies are needed to clarify the mechanism of monozygotic splitting, especially the effects of IVF treatment on early embryo development.

## Data Availability

The datasets analyzed during the current study are available from the corresponding author on reasonable request.

## Abbreviations

AH: Assisted hatching
ART: Assisted reproductive technology
CC: Clomiphene citrate
DC-DA: Dichorionic-diamniotic
FET: Frozen embryo transfer
FSH: Follicle-stimulating hormone
GDM: Gestational diabetes mellitus
GnRH: Gonadotropin-releasing hormone
hCG: human chorionic gonadotropin
hMG: human menopausal gonadotropin
HRT: Hormone replacement treatment
ICSI: Intracytoplasmic sperm injection
IVF: In vitro fertilization
MC-DA: Monochorionic-diamniotic
MC-MA: Monochorionic-monoamniotic
MZT: Monozygotic twinning
PCR: Polymerase chain reaction
PCOS: Polycystic ovarian syndrome
PGT: Preimplantation genetic testing
SNM1: Survival motor neuron gene
WGA: Whole genome amplification
